# Strengthening prehospital clinical practice guideline implementation in South Africa: a qualitative case study

**DOI:** 10.1186/s12913-020-05111-x

**Published:** 2020-04-24

**Authors:** Michael McCaul, Taryn Young, Stevan R. Bruijns, Mike Clarke

**Affiliations:** 1 Centre for Evidence-based Health Care, Division of Epidemiology and Biostatistics, Department of Global Health, Stellenbosch University, Cape Town, South Africa; 2 Division of Emergency Medicine, University of Cape Town, Cape Town, South Africa; 3 Centre for Public Health, Queen’s University Belfast, Belfast, Northern Ireland UK

**Keywords:** Guidelines, Prehospital, Qualitative, Case study, South Africa, Emergency medicine, Paramedic, Recommendations, Guideline development, Guideline adaptation

## Abstract

**Background:**

Methods on developing new (de novo) clinical practice guidelines (CPGs) have received substantial attention. However, research into alternative methods of CPG development using existing CPG documents (CPG adaptation) — a specific issue for guideline development groups in low- and middle-income countries — is sparse. There are only a few examples showcasing the pragmatic application of such alternative approaches in settings with time and budget constraints, especially in the prehospital setting. This paper aims to describe and strengthen the methods of developing prehospital CPGs using alternative guideline development methods through a case study design.

**Methods:**

We qualitatively explored a CPG development project conducted in 2016 for prehospital providers in South Africa as a case study. Key stakeholders, involved in various processes of the guideline project, were purposefully sampled. Data were collected from one focus group and six in-depth interviews and analysed using thematic analysis. Overarching themes and sub-themes were inductively developed and categorised as challenges and recommendations and further transformed into action points.

**Results:**

Key challenges revolved around guideline implementation as opposed to development. These included the unavoidable effect of interest and beliefs on implementing recommendations, the local evidence void, a shifting implementation context, and opposing end-user needs. Guideline development and implementation strengthening priority actions included: i) developing a national end-user document; ii) aligning recommendations with local practice; iii) communicating a clear and consistent message; iv) addressing controversial recommendations; v) managing the impact of interests, beliefs and intellectual conflicts; and vi) transparently reporting implementation decisions.

**Conclusion:**

The cornerstone of a successful guideline development process is the translation and implementation of CPG recommendations into clinical practice. We highlight key priority actions for prehospital guideline development teams with limited resources to strengthen guideline development, dissemination, and implementation by drawing from lessons learnt from a prehospital guideline project conducted in South Africa.

## Introduction

The methods for creating de novo (new) clinical practice guidelines (CPGs) have been well developed and are supported by numerous examples and standards [1–3]. However, de novo CPG development is often an expensive, time consuming, and human resource-intensive process that is out of reach for guideline development groups in resource-poor settings. As such, various alternative methods for CPG development have been proposed (termed CPG adaptation). These methods avoid re-inventing the wheel by drawing on existing up-to-date high quality CPGs to make recommendations that are locally applicable [4–10]. However, relative to de novo methods, there is a paucity of pragmatic case studies, specifically in prehospital care for developing guidelines or protocols. Displaying the application and challenges of such methods in resource-constrained settings is important, especially considering the attractiveness of adaptation methods due to cost and time savings [11].

One reason for this is that adaptation methods are still underused in prehospital care. A 2018 landscape analysis of 276 prehospital CPGs indicated less than 8% of CPGs used adaptive methods, with less than 2% of all CPGs originating from low-to-middle income countries (LMICs) [12]. This resulted in a call by the authors to showcase pragmatic applications of adaptive guideline development methods in resource-poor settings for prehospital care and to develop capacity for local guideline developers to use adaptation methods [13]. Supporting this, guideline developers have an expanding pool of up-to-date high quality international prehospital CPGs that can be adapted for their local setting, using a variety of methods. Furthermore, research and guideline development for prehospital care is unique due to the emergency setting, making trials difficult, adding to the general paucity of prehospital evidence. The prehospital setting is also varied across countries, ranging from informal first aid responders to doctor-lead helicopter services, making generalisability of evidence problematic.

Most alternative methods follow similar steps to de novo development, except they draw on existing high-level evidence (CPGs or systematic reviews) to develop recommendations, instead of doing their own syntheses of primary level evidence in new systematic reviews. Examples include adopting, adapting, or contextualising guideline recommendations to a local setting, which has successfully been implemented in LMICs [11, 14]. Other methods simply fast track or remove certain steps, as proposed by the G-I-N accelerated guideline working group, or adapt existing CPGs (The ADAPT process) [15, 16]. Schunemann et al (2016) developed a process of incorporating the GRADE Evidence to Decision (EtD) framework in developing CPGs from existing systematic reviews [8]. Despite these various methods, examples are rare and very few have been described in prehospital care, with most CPGs in this context still being developed de novo, predominantly in high-income settings [11].

Most examples of alternative guideline development methods in prehospital care use a hybrid combination of synthesising primary evidence (de novo methods) and adaptation methods [12]. Globally, these methods are poorly described, which is not surprising as quality reporting criteria for alternative guideline development methods do not exist, although work is in progress [17]. Even AGREE II, a guideline quality appraisal tool, does not make provision for adapted CPG methods [3].

Guideline implementation is an essential part of the guideline process, with unique challenges and barriers, which are often context specific [18, 19]. Furthermore, in allied health, and especially in the South African prehospital setting, there is uncertainty regarding who is responsible for implementing guidelines and how this should be done [18]. Additionally, South Africa has no national guideline coordinating centre, limiting standardisation of guideline development and implementation. In order to strengthen guideline uptake, the barriers and challenges to both guideline development and implementation should be explored.

There is a clear need to describe alternative guideline methods thoroughly and describe challenges and solutions, specifically using examples relevant for resource-poor settings (e.g. any setting where funds, capacity or expertise is limited), whether from high or low-to-middle income countries. This paper helps to fill this gap by describing the methods and challenges of developing and implementing prehospital CPGs using alternative guideline development methods through a case study design.

## Methods

### Study design

We qualitatively explored a CPG development project conducted in 2016 for prehospital providers in South Africa as a case study. This case study aims to strengthen CPG development in low resource settings by presenting an in-depth understanding of the case, particularly by describing the methods, processes, barriers, challenges, and solutions of the case. Intrinsic case studies intend to illustrate and detail a unique case within a bounded system and are appropriate when intending to develop an in-depth understanding and analysis of a clearly defined project [20]. We purposefully sampled a single guideline project, led by the African Federation for Emergency Medicine (AFEM), and key role-players in the project. The COREQ (Consolidated criteria for reporting qualitative research) statement, the current gold standard in qualitative research reporting [21], guided our research and write up.

#### The case: African Federation for Emergency Medicine CPG project

The Health Profession Council of South Africa Professional Board of Emergency Care (HPCSA PBEC) awarded a bid to revise the current emergency care protocols to the AFEM, collaborating with the Centre for Evidence-based Health Care (Stellenbosch University) and the Department of Emergency Medical Sciences (Cape Peninsula University of Technology) in late 2015. The final CPG was submitted to the HPCSA PBEC in June 2016 [22]. The project’s mandate was to develop a contextually appropriate CPG for prehospital care in South Africa that is patient centred, based on best evidence, and aligned to the current and future prehospital educational bands [23].

This case study is set within this AFEM project, methods described previously [11], where the temporal boundaries of the case start with drafting a collective bid to develop the prehospital CPG (in early 2015) and ends in middle to late 2017, approximately one year after submission of the guideline to the PBEC. Key stakeholders in the project include members of the guideline panel, the PBEC, the project guideline methodologists, and advisory panel members. The case boundaries are set wide so that a holistic case can be presented, taking into account all aspects of the case including topics considered outside of the project’s original mandate such as guideline dissemination and implementation.

#### Participants

Key informants were purposefully sampled in order to maximize the diversity of data relevant to the study aims. We invited participants from the guideline funders (*n* = 1), core guideline panel (*n* = 4) and the guideline advisory board (*n* = 6) via email or telephone. Unfortunately, the guideline funders (HPCSA PBEC), due to certain regulatory processes in relation to ongoing stakeholder engagement, were unable to contribute further to this research project (MM 2019, personal communication, 19 August 2019) and, thus, our sample comprises a total of 10 participants. Supportive material included the AFEM guideline document, guideline panel meeting minutes, and interview and focus group notes.

#### Data collection

We collected and integrated various forms of qualitative data, from focus groups and in-depth interviews to meeting notes and case documents for an in-depth understanding of the case. Interviews were conducted during March and April 2019 in boardrooms or venues appropriate for the participants, such as personal offices or over Skype. Each interview lasted approximately 40 min. A focus group was conducted for the core guideline panel to enrich the depth of the data. Only participants and investigators were present during the in-depth interviews and focus groups. An independent, experienced qualitative researcher (KG, Extraordinary Professor, PhD) facilitated the focus groups and interviews as the primary investigator (MM) was involved with the CPG development as a guideline panellist. He (MM, Senior lecturer, MSc) acted as the scribe and was present during the in-depth interviews to take notes and manage recordings. At focus groups, an informal conversational atmosphere was promoted. During focus groups, participants faced each other in a circular boardroom arrangement, to promote a relaxed and comfortable atmosphere. Focus group and individual interviews were recorded electronically and transcribed verbatim for analysis. Transcripts were returned to participants for comment (member checking) and adjustments incorporated. Data saturation was discussed among the author team.

Data were collected via a semi-structured interview schedule (Additional file 1) for individual in-depth interviews and focus groups. Since the guideline project occurred in late 2016 and some participants might suffer from recall bias, participants were emailed the final CPG and some recent publications around the guideline, describing processes and factors leading up to the guideline and beyond as a terms of reference document [11, 13, 24, 25]. They were also sent the overarching topics for potential discussion, to help them prepare. As such, participants were aware *a priori* of the research and reasons for doing the research.

#### Data analysis

Transcribed data were analysed thematically by MM with an inductive approach through manual coding [24]. Codes and themes were discussed and reviewed among the author team. All transcripts were read as a whole to familiarise the analysts, followed by a process of condensing verbatim text into condensed meaning units. Next steps involved labelling condensed meaning units by formulating codes and then grouping these codes into categories. Where appropriate, with sufficient data depth (and higher levels of abstraction), categories merged into themes, and across themes into overarching themes. Themes and overarching themes were presented graphically and grouped within the adaptive CPG development process [11], similar to a coding tree (Additional file 2). Themes originating outside of a guideline development framework were still reported and coded.

#### Trustworthiness and reflexivity

In this study, we sought to ensure that the research process was trustworthy, so that the findings could be considered a credible reflection of reality [25]. Several measures were taken to establish credibility, dependability, confirmability, and transferability. These included peer scrutiny of the project, data and analysis, description of study context, debriefing sessions, independent experiences of facilitators for interviews and focus groups, iterative questioning, purposeful sampling, rich use of quotations from participants, member checking, and reflection of research beliefs and assumptions.

Throughout the study, we attempted to adhere to the methodological principle of reflexivity [25]. The principle investigator (MM) has a background in prehospital emergency care and was involved as a methodologist in the AFEM Emergency Medical Services (EMS) CPGs as a core guideline panel member. During analysis, MM drew from his lived experiences as an AFEM guideline panel member and past guideline research [11–13, 22, 23] to explore the latent meaning of text. However, as noted above, focus group and interviews were facilitated by an independent experienced researcher with MM acting as a participant during a focus group (as an AFEM guideline panel member). This linkage meant that most participants were aware of the research.

#### Ethics

Ethics approval was obtained from the Stellenbosch Faculty of Medicine and Health Sciences ethics committee (S17/03/069). Written informed consent was obtained from participants.

## Results

### Participants

We conducted six in-depth interviews and one focus group (*n* = 4). All participants were involved in the AFEM guideline project and represented various facets of the guideline process. These include the advisory boards, core guideline panel, and project management. Due to the relatively small size of the project, further details of participant characteristics cannot be detailed, to protect anonymity. Unfortunately, the funders of the project were not available for interviews.

### Results overview and themes

Due to the inductive nature, emerging themes centred on existing challenges and potential solutions to strengthen prehospital guideline development and implementation. This is reflective of the current context of the guideline process, where the primary concern is guideline dissemination and implementation, as opposed to guideline development.

**Fig. 1:**
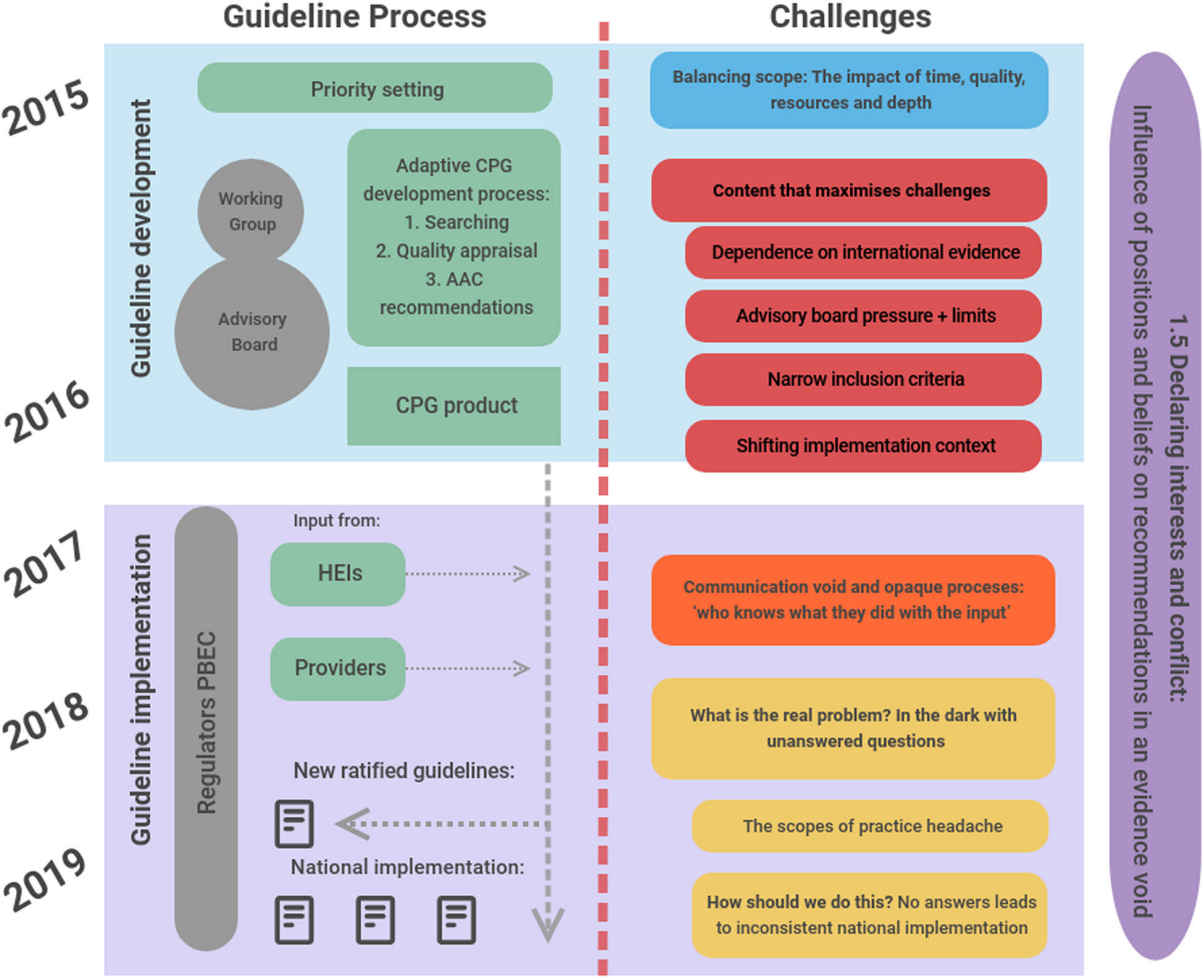
AFEM Guideline development and implementation process with linked challenges

**Fig. 2:**
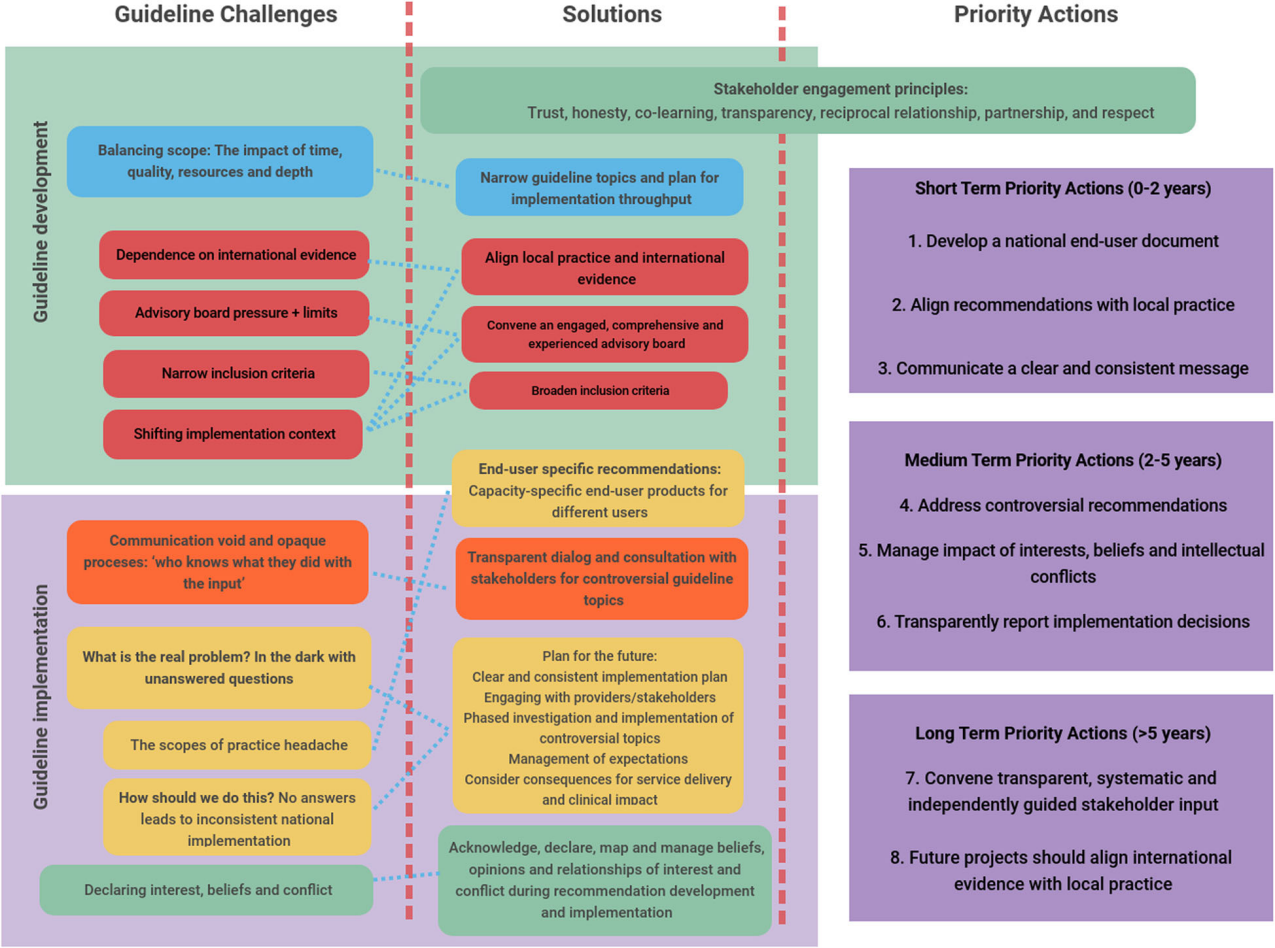
Guideline challenges, solutions and priority actions

Overarching themes emerging from the data are grouped according to the guideline development process as shown in Fig. 1, namely challenges, recommendations, and priority actions. Themes are not mutually exclusive, as there was often overlap. However, this grouping aids in unpacking the bigger picture, exploring relationships and describing the larger narrative within the case study boundaries. Results are summarised and presented according to the challenges linked to the chronological guideline processes (Fig. 1) and eventually to priority actions for guideline development and implementation (Fig. 2).

### Successes

#### CPG and scope of practice impact: majority benefited with expanded access and care options

Participants felt that the CPGs produced positive change to prehospital care in South Africa, by updating outdated clinical practice to the vast majority of providers, advocating access to improved medicines and interventions by guiding policy change and enabling curriculum updates for new providers. This was especially true for example ‘*around non-controversial topics like fluid administration’* and interventions or practices relevant to the majority of providers, including basic ambulance assistants and intermediate life support providers. The guidelines were also the first of their kind, advocating clinical practice based on the synthesis of the best available evidence, replacing decades-old practice and advocating for change.

### Challenges

Producing a prehospital CPG using adaptation methods was not without challenges. We unpack these challenges below, many of which are linked to guideline processes and timestamps presented in Fig. 1, where a distinction is made between challenges experienced during guideline development versus guideline implementation.

#### Balancing scope: the impact of time, quality, resources and depth

In this overarching theme, we explore the factors (seen as challenges) that impacted the CPG scope. Each factor is represented as a corner within an ‘iron pentagon’ (term adapted from the project management triangle), where some factors in this case study were considered constant such as budget or deadlines, creating tension in factors that can vary, like guideline scope, depth, and even quality.

A particular challenge was balancing guideline scope, which has potential to vary, with unyielding factors such as project deadlines and budget. This was a difficult space to navigate for the guideline panel, because there was competition between maximising guideline scope and quality with available resources, depth and time. ‘*We [couldn’t] deal with everything, we only had 8 months’,* as *‘scope was a double-edged sword: whatever we didn’t address, there would just be a void in practice’*. The panel was thus ‘*forced to address as many topics as [it] could, but still produce a quality product’.* In the end, panellists believed that priority topics were sufficiently covered, and clear gaps were identified for future research. However, maximising scope was not without consequences, as resources and time could have been spent on other challenges.

#### Context that maximises challenges

This overarching theme highlights the challenging and shifting context within which the guideline was developed and eventually implemented. A useful baking metaphor emerged in which the AFEM guideline context can be thought of as the recipe to maximise challenges: by adding a handful of local evidence gap, mixing in a shifting implementation context, baking with strict methods, and serving to opposing end-user needs. The first emerging theme was the guideline development group’s dependence on international evidence, because there was no high quality, up-to-date local CPG to draw from:

*‘We were very dependent on international evidence. There’s not a lot that we could find that’s locally of high quality that we could include that would inform clinical practice.’*

This resulted in a substantial number of recommendations that needed to be adapted or contextualised to the local setting, as opposed to being adopted. This placed added pressure on the advisory board, who had little to no experience in CPG development or adaptation.

Due to the strict *a priori* inclusion criteria, which prioritised high-quality CPGs over other guidance documents such as algorithms or protocols, the process inadvertently excluded evidence that would have been useful to inform end-user document designs.

*‘It’s not the best evidence but I think a lot of the stuff that was excluded, may have actually been helpful to inform local practice.’*

Lastly, during the guideline development years *‘the whole profession* [prehospital emergency care providers] *was shifting from a short course-based system to a professional degree-type practitioner, where we have a technician to practitioner shift’,* which complicated and directly impacted on how the CPGs have been implemented and received as industry transitioned away from training skills based short course prehospital providers.

#### Communication void and opaque processes: *‘who knows what they did with the input’*

This overarching theme stems from the perceived communication vacuum during the guideline implementation phase by regulators.

Once the CPGs were handed over, participants described a ‘*vacuum of communication from the board … while clearly some internal board processes [were] going on’.* This was linked to opening the CPGs for comments from institutional and operational services and eventually the public: *‘ … but then again, a complete communication blackout while they considered that info presumably, but who knows what they did with the input. There is no transparency in how they took our recommendations and how it ended up with their scope of practice recommendations and having had a basically a communication void for eighteen months’*. This theme was reflected across interviews, the concern linked to the lack of communication and transparency of the decision making process, the communication void harming the paramedics, and the autocratic style of dissemination: *‘ “thou shalt do this”, without engaging with the frontline stakeholders’*.

#### Implementing CPG recommendations: in the dark with unanswered questions

This overarching theme comprises four sub-themes unpacking the central issues faced within and beyond the AFEM guideline development process.

##### What is the real problem? The ‘scopes of practice headache’

A prevailing trend noted across interviews was the notion that the AFEM CPG, itself with recommendations, was not the inherent problem or issue for industry. Rather, the translation of the CPG recommendations to scope of practice (for implementation) for varying cadres of prehospital providers was described as the true ‘*headache’,* as *‘we don’t have a problem with the evidence based statements* [referring to the guideline output] *… the problem is how the professional board* [regulator] *has interpreted some of those statements and converted them into new scopes of practice’*.

The core problem however, is not so simply explained. It is extensively complex, highlighted by various sub-themes, such as i) the paramedic and academic disconnect: the need for understanding both ways; ii) the impracticality of engaging with the majority of providers; iii) project resource and budgetary restraints; iv) lack of implementation evidence; and v) industry maturity and lack of research experience.

Importantly, the *‘scopes of practice headache’* ‘*mostly negatively affected a small group of well-educated and vocal people, which completely undermined the whole implementation’*. In contrast, the CPGs’ recommendations and scope of practice changes impact have been overwhelmingly positive, *‘as the majority of registration categories* [paramedics] *have benefited, as they have been given an expanded scope of practice*’ and *‘improved access’* and *‘forced needed change’* to decades-old protocols.

##### How should we do this? No answers lead to inconsistent national implementation

Another key sub-theme is the lack of a timely, practical implementation strategy from regulators or the national department of health as *‘there is still a lot of confusion* [re implementation]*’*. These implementation challenges led to two sub-themes:

#### Unguided national implementation and end-user documentation: rising provincial training variation and provider ‘upskill’ exploitation

The lack of a national implementation plan and single end-user document for all provinces has led to standardisation concerns as *‘each province has added its own strategy of interpreting and operationalising the guidelines’*. Moreover, there are concerns that paramedics, especially basic providers, will be exploited financially by unregulated short course training opportunities *‘charging exorbitant fees if you want to upgrade’*.

#### Overwhelmed institutions and empty coffers lead to rote learning

This sub-theme specifically applies to qualified short course paramedics, where service providers and public training institutions are overwhelmed by the training impact of upskilling industry to the new scopes of practice. This raises various concerns and effects such as *‘cost implications for new equipment’,* trainer to provider ratio imbalance, and the lack of sufficient short course training time to accommodate the expanded scope of practice.

*‘It is overwhelming for the time frame to fit the actual teaching and training of the new scope of drugs … teaching them and getting them to understand, we have a huge problem.’*

In summary, we unpacked the real issues, challenges and downstream effects experienced in the AFEM project, catalysed by a non-existent implementation plan from regulators, and pressured service providers and educational institutions with limited budgets.

#### Declaring interests and conflict: influence of positions and beliefs on recommendations in an evidence void

In the AFEM guideline development process, conflicts of interest were handled through standard methods, by means of a conflicts of interest declaration and recording appropriate judgements if potential interests or conflicts arose. However, none did. Our data revealed that predefined beliefs, interests, history, positions, and relationships between individuals and organisations have a far greater influence on final recommendations and, specifically in this case, how they are implemented in practice, than anticipated. The theme deals with three issues: i) conflicting beliefs because of previous knowledge; ii) conflict because of knowledge of resource constraints and what best evidence is; and iii) questioning authority.

For example, one participant highlighted the influence of their own predetermined beliefs on controversial topics and noted that their *‘mind about the evidence was often already made up’*. Concerns of beliefs and conflicts of interest was often centred around controversial topics such as intubation and its implementation, as opposed to uncontroversial topics such as ‘*who can administer aspirin for heart attacks*’. Additionally, for implementation decisions, the lack of local evidence to objectively support implementation decisions had a drastic impact on what influenced decisions, as there was *‘not enough [evidence] to sway opinion’*, and thus previously held beliefs and positions around scope of practice, for example, influenced decisions.

When drafting recommendations from an advisory board perspective, managing conflict of interest was also described as an internal struggle between the recommendations (from international evidence) and what is practical in South Africa, as noted here:

*“My biggest conflict of interest if you want to call it that, was knowing what is available and what is not available and what is practical and trying to reconcile that with the scientifically valid statement [recommendations], even if you don’t entirely agree with it”.*

The concerns that advisory board members, as experts in their field, *‘all come with their own bias’,* and that without experts the guideline validity would be questioned as *‘a wider audience of end-users is going to say, “what do they know?”’,* were highlighted as being problematic. This can be described as a catch 22 or dilemma, where guideline validity is questioned if experts are not involved but biased if experts are involved, highlighting the importance of acknowledging the hidden influence of belief and interest in guideline development.

Overall, the challenge is managing beliefs and conflicts of interest, as this participant stated: ‘*the notion of belief was not well managed’.* This is an overarching concern voiced by participants, especially during guideline implementation discussions and times of discourse during the project.

### Recommendations and solutions

Building from challenges identified, we present various recommendations to strengthen guideline development and implementation, which arise from this AFEM CPG case study.

#### End-user specific recommendations: balancing guideline delivery with paramedic capacity

During the AFEM guideline development process, the wording of recommendations was kept as close as possible to the original adapted or adopted CPGs. This overarching theme deals with how recommendations are worded, and how they should be translated to end-user content. The theme has two sub-themes: i) flexible versus prescriptive recommendations; and ii) the need for capacity-specific recommendations for different user levels.

##### Flexible versus prescriptive recommendations

This sub-theme explores the notion of flexible recommendations, for the ‘*intensely trained’* paramedic, where less prescriptive wording for recommendations was advocated. More prescriptive wording was suggested for lower levels of providers as described below:

*“Lower levels I think must be given very prescriptive guidelines. You must give oxygen if the child has recession.”*

However, controversially, it was noted that paramedics “*need room to deviate, see what works, see what doesn’t work”*; paramedics thus need to be given room to deviate as “*harm could be caused if you’re too prescriptive”.*

##### Capacity-specific recommendation for different user levels

Participants suggested different styles of recommendations and how they are presented, depending on the different users. One participant presented it as an analogy, contrasting the in-hospital to prehospital paradigm noting, *“as much as we want our paramedics to be thinking paramedics, we want them to be thinking within a defined paradigm, whereas in the hospital your paradigm is much wider or much broader”.*

In summary, this overarching theme speaks to the need for creating provider-centric recommendations, and provider-specific end-user content appropriate to the providers’ capacity and training, as noted during the focus groups:

*“The upper echelon needs the CPGs, they need to have more freedom. The lower echelon needs more structured protocols”.*

#### Align local practice and international evidence: where is the evidence coming from?

Linking closely with the project’s dependence on international evidence due to a local evidence void described in challenges, here we explore an emerging downstream solution. In this overarching theme, we unpack the conundrum of developing local recommendations with foreign, international evidence and present a solution.

Participants expressed the notion that in emergency care *‘international evidence is sometimes the only evidence there is’*, and that local robust evidence is lacking. Some noted that there needs to be alignment of recommendations with local practice to be *‘cognisant of where the research has been done … and to contextualise’* to minimise the evidence to practice gap of *‘this is not what we do locally’*. Practical examples were given to emphasise the point, including alignment of adopted World Health Organisation recommendations for treating dehydration in children and alignment of local guidance stipulated in South Africa by the Western Cape Provincial Department of Health. This theme then leads to an appropriate segue for guideline implementation solutions.

#### Plan for the future: Deal with controversies, focus on follow-through, not breakthrough

Even if recommendations are appropriately contextualised and aligned to local practice, recommendations can only drive uptake so far; follow-through to implementation is needed to ensure practice change. This overarching theme unpacks guideline implementation concepts and potential consequences of implementation forethought.

##### Implementation strengthening concepts

Various suggestions were noted for future and current guideline implementation efforts. Foremost is dealing with controversies, *‘like solving the airway management question that’s forever thrown out … ’*, and then focusing on filling focused priority gaps via a de novo process with appropriate implementation follow through, as stated by a guideline panel group member:

*‘The step is to look at the gaps and I would say to do systematic reviews on those gaps, appropriately synthesise, and then take it through a guideline panel process. Similar to what we did, but for very focused questions and then come to a very clear recommendation with a clear implementation plan that can set the record straight for those priorities, even priorities where there’s a lot of implementation issues … ’*.

In light of follow through, various implementation and dissemination strengthening categories emerged, enabled through providing clear communication. These included: i) providing a clear and consistent implementation plan; ii) engaging with providers via roadshows or similar activities; iii) phased investigation and implementation of controversial topics; and iv) management of expectations in light of change resistance.

Despite the new CPGs having been implemented, albeit with challenges, these solutions may prove useful for current and future efforts.

##### Consequences of implementation forethought

As noted in challenges, lack of implementation foresight was clearly a prominent concern. In this theme, we describe potential downstream consequences. A resonating concern from participants was that *‘removing skills is going to impact on our patient care and our service delivery* [often referring to intubation]*’*, similarly with related examples of unequitable service delivery effects and consequences such as seen for interhospital transfers of neonates:

*‘People are going to refuse to take babies from a district to a higher level, because now the same person who came last month can’t come this month, because of new rules and regulations [referring to scope changes]. What’s going to happen to the baby? The baby is going to die or become very damaged’* (Clinician, Advisory board member).

In summary, this overarching theme highlights the importance of evidence-based recommendations that are accompanied with an implementation ‘follow through’ plan, because use of evidence without implementation will most likely lead to harm.

#### Plugging the implementation conversation gap: open, transparent and broad dialogue

Having identified specific sequential challenges, solutions emerged to fill implementation gaps, which were specifically related to ‘*three, maybe four controversial topics’*. Solutions included *‘addressing those [topics] openly’*, broader engagement and input from experts, national department of health and regulators, and lastly agreement from stakeholders like a *‘joint legal minute … then at least it’s transparent when the scope of practice comes out’*.

In summary, this overarching theme promotes timely communication and an open, transparent decision making process with broad engagement and agreement from stakeholders for informing national implementation decisions.

### Priority actions

Based on the overarching challenges and recommendation themes that emerged from our case study, the following priority actions are recommended, summarised in Fig. 2:

#### Short term priority actions (0–2 years)

- Regulators and the National Department of Health, together with stakeholders, should develop national end-user-specific guidance documents which are reviewed by an independent academic body;
- Align guideline recommendations with local practice and guidance to strengthen guideline uptake and the continuum of care through contextualisation or adaptation;
- A clear, obtainable and phased implementation strategy should be communicated by regulators and the National Department of Health to guideline end-users and stakeholders with opportunity for two-way dialogue and collaboration.

#### Medium term priority actions (2–5 years)

- Controversial prehospital recommendations (e.g. rapid sequence intubation) and guidance gaps should be updated and revised using transparent decision support tools (e.g. EtD) with effective implementation as an end goal;
- The impact of interests, beliefs, relationship, and intellectual conflicts must be managed when considering how CPG recommendations are developed and implemented during stakeholder engagement and input;
- Decision makers should transparently report implementation decisions to guideline end-users, detailing processes, involved stakeholders, conflicts and interests, and areas of disagreement.

#### Long term priority actions (> 5 years)

- Future prehospital guideline development projects should align international best evidence with local guidance;
- Convene transparent, systematic and independently guided stakeholder input.

## Discussion

True to the context and current issues faced by paramedics and stakeholders in South Africa, our results focused around unpacking the pressing challenges and linked solutions, as opposed to describing past methods, described previously [11]. Our results speak predominantly to the guideline steps after recommendations have been finalised, when decisions are made as to how recommendations are operationalised in clinical practice. Steps 14 (Wording of recommendations and of considerations about implementation, feasibility and equity) and 16 (Dissemination and implementation) detailed in guidelines 2.0 by Schunemann expand on these concepts, but provide little insight on how to navigate stakeholder engagement regarding implementation once recommendations have been developed in order to maximise local guideline uptake [26]. This critical juncture, the transition from evidence-based recommendations to contextually appropriate and pragmatic decisions for clinical practice and target-users, is where stakeholder engagement broke down and where further work is needed. We noted how competing interests, whether intellectual, financial or indirect (relationships or beliefs) need to be acknowledged and managed transparently, especially when engaging stakeholders and when making implementation decisions.

*A priori* acknowledgement and documentation of beliefs, intellectual conflicts, relationships and interests of all stakeholders, including guideline implementers, during guideline development and specifically implementation is essential. Doing so could have prevented various challenges for the AFEM guideline group. However, this is an international challenge, where strategies such as the G-I-N nine principles for managing conflicts in guideline development are continuously being updated, to address disclosure and management of competing interests [27, 28].

This is specifically important for controversial issues, where evidence and implementation strategies are often unclear, as in our case study. For these issues, when decisions around operationalisation of recommendations are made, transparency in decision-making, and management of interest and conflict is of utmost importance, as reflected by the International Committee of Medical Journal Editors updated policy on competing interests [29]. Guidelines are particularly vulnerable to the effects of conflicts and interests, due to stakeholder engagement being a cornerstone guideline process [30]. The guideline community is setting new quality and evidence thresholds [17]; however, in considering evidence, guideline groups must consider the appropriateness of evidence. This is especially true for CPGs using adaptation methods, where an assessment of the generalisability and acceptability of evidence to context and guidelines users is often absent in dialogue. Thus, even in such challenging dialog it is paramount transparency of decisions is held, especially in the face of conflicts of interest. Furthermore, we identified various universal themes this case study experienced across jurisdictions and health care challenges such as scope of practice issues, boundaries of implementation and overwhelmed institutions [18, 19, 31–33].

Assisting with this vulnerability, the GRADE EtD framework is a useful primer for controversial issues, as guideline panellists transparently document and deal with issues such as feasibility, acceptability, resources and equity, with the EtD process ending only when a recommendation has been ratified [34]. However, useful CPG adaptation examples exist from LMICs, showcasing various methods of strengthening guideline uptake by considering local issues either through qualitative research or stakeholder engagement during and after recommendations have been drafted [33]. However, evidence is till inconclusive whether CPG adaptation methods are superior to de novo methods. Often time and cost is cited as advantageous for CPG adaptation, however stronger evidence is needed with equivalent comparisons or better insight into different contexts and their available resources. Useful lessons can be adopted for future prehospital projects in creating fit-for-purpose and efficient CPGs, such as conducting a contextual analysis and integration of end-user needs into guideline recommendations [7] or using hierarchical search strategies [6]. Other *a priori* solutions include establishing the rationale for engaging stakeholders, identifying stakeholder communities, how engagement will work (roles and modes), and importantly what conflicts of interest procedures and conflict management resources are needed [35], of which various exist [27, 28, 34]. Furthermore, when considering implementation decision domains such as acceptability or feasibility, qualitative evidence synthesis, a research area lacking in emergency medicine and prehospital care, should be considered [36].

For the South African EMS setting, we recommend a phased implementation approach, showcased in allied health stroke guidelines, where an ideal timeline is linked to recommendations that cannot be adopted immediately [10]. This would be useful for controversial and complex interventions such as intubation, prehospital thrombolysis or scalp vein cannulation for infants. We further recommend, for the South African EMS and similar settings, creating end-user specific documents, such as strict protocols for short course trained paramedics and more flexible guidance documents for higher qualified paramedics, of which useful examples exist in primary health care for nurses [37, 38] and emergency medicine [39].

It is refreshing to see progress being made by the HPCSA PBEC, which has started addressing prehospital end-user needs and challenges highlighted previously [22, 40] by regulating paramedic CPG updates, releasing a CPG FAQ [41] and seeking approval of new medicines and interventions. However, in order to equip paramedics to make decisions based on the best available evidence, all national decision makers will need to engage in collaborative action, where short to long term priority actions provide guidance.

This study has a key limitation: we were unable to interview the HPCSA PBEC, which would have provided valuable insight into the South African regulatory framework and implementation challenges from their perspective. Although the National Department of Health was not part of the bounded AFEM case, its role in downstream implementation to date, including the HPCSA PBEC, is an essential perspective and future research exploring the recommendations to implementation gap should incorporate these stakeholders. Additionally, our research reflects the perceptions and thoughts of an influential but relatively small group of people, each with their own agenda and biases. Furthermore, our research does not shed light on incorporating patient perspectives for prehospital guidance but rather on engagement of guideline end-users and decision makers.

## Conclusion

The cornerstone of a successful CPG development process is the translation and implementation of CPG recommendations into clinical practice. We highlight time-sensitive priority actions for prehospital guideline development or adaptation teams, national departments of health, regulators and the prehospital industry in South Africa to strengthen guideline development, dissemination and implementation by drawing from lessons learnt from the AFEM prehospital guideline project. We also highlight challenges during stakeholder engagement when implementing guideline recommendations. These need to be addressed if guideline uptake and implementation is to be strengthened.

## Supplementary information

**Additional file 1.** Semi-structured interview schedule (Example Interview 1).

**Additional file 2.** Alternative guideline development process.

## Data Availability

As this study analyses qualitative data and participants did not consent to have their full transcripts made publicly available, data excerpts are available from Stellenbosch University Research Data Repository for researchers who meet the criteria for access (https://scholardata.sun.ac.za/).

## Abbreviations

AAC: Adapt, adopt or contextualise
AFEM: African Federation for Emergency Medicine
AGREE II: Appraisal of guideline research and evaluation
COREQ: Consolidated criteria for reporting qualitative research
CPG: Clinical practice guideline
EMS: Emergency Medical Services
EtD: Evidence to Decision
G-I-N: Guidelines International Network
HEI: Higher Education Institution
HPCSA: Health Professions Council of South Africa
LMICs: Low- and middle-income countries
PBEC: Professional Board of Emergency Care
