# Supplementary material for: Strengthening prehospital clinical practice guideline implementation in South Africa: a qualitative case study
Source: BMC Health Serv Res. 2020 Apr 24;20:349. doi: 10.1186/s12913-020-05111-x (PMC7183123; doi:10.1186/s12913-020-05111-x)
Supplement: Supplementary file 1 — Additional file 1. Semi-structured interview schedule (Example Interview 1). [file 12913_2020_5111_MOESM1_ESM.docx]

## Additional file 1: Semi-structured interview schedule (Example Interview 1)

| De Novo approach | AFEM adaptation approach | Focus | Example Probing Questions |
| --- | --- | --- | --- |
| 1. Organisation, budget, planning and training | * |  |  |
| 1. Priority setting | * |  | Reflecting on the current priority areas in the pre-hospital guidelines do you believe they have addressed the right priorities? Are there any gaps? |
| 1. Guideline group membership | Include advisory board (clinical and methodological) |  | Reflections on the membership of the advisory group. Did the composition of the advisory group impact on the uptake of the guidelines by paramedics? What was missing re membership or representation on the advisory board? |
| 1. Establish guideline group processes | Include decision framework for using existing guidelines and recommendations. |  | Can we touch on your perceptions of the guideline development process? Can you reflect on the process of progressing international evidence to recommendations? And now on the local implementation process? |
| 1. Identify target audience and topic selection | * |  | Any comments on how the topics for the guideline was established? Recommendations for future topics? Gaps? |
| 1. Consumer and stakeholder involvement | * |  | Paramedics who will use this guideline.  Have they been engaged sufficiently to enable them to take up guideline?  ??Communication??  Can you reflect on the engagement and involvement of consumers and end-users of the guidelines? Do you believe this could have been done better? How? |
| 1. Conflicts of interest | * |  | Do you think CoI were appropriately handled?  What can you offer about declaring interests? |
| 1. Question generation | Create broader questions that are transferable to key priority areas applicable and likely to be reported in guidelines |  |  |
| 1. Considering importance of outcomes and interventions, values, preferences and utilities | * |  |  |
| 1. Deciding what evidence to include and searching for evidence | Clearly defining inclusion of high quality, up-to-date guidelines and perform comprehensive searches including guideline clearinghouses, Google and traditional databases |  | Can you comment around the process that was taken using international guidelines and current best evidence to inform the local guideline writing? |
| 1. Summarizing evidence and considering additional information | Mapping evidence and/or guidelines by priority areas and/or questions |  |  |
| 1. Judging quality, strength or certainty of a body of evidence | Using AGREE II appraisal for guidelines and ranking included guidelines by date, relevance and overall quality |  |  |
| 1. Developing recommendations and determining their strength | Adopting, adapting or contextualising guidelines  Extract recommendations relevant to priority areas and questions  Reviewing adopted, adapted or contextualised recommendations with advisory boards |  | Can you give me your opinion of the way the recommendations were written, and the evidence strength statements underpinning them? |
| 1. Wording of recommendations and of considerations about implementation, feasibility and equity | Reporting original working of recommendations levels of evidence and/or strength in plain language  Considering implementation points and practice points for each recommendation that has been adopted or contextualised |  | Wording assisting in end-user uptake in local contexts?  How well was the system prepared to take on the changes related to evidence-based recommendation implementation?  What recommendations could you make to get implementation underway?  What lessons have you learnt with implementation? |
| 1. Reporting and peer review | * |  | How well were the guidelines peer-reviewed? How well was industry engaged in feedback to the guidelines? |
| 1. Dissemination and implementation | * |  | Can you comment on the way the guidelines were originally disseminated? Could this have been done better?  There has been some time period which has happened between dissemination and implementation. Do you have ideas about how this can be moved along? |
| 1. Evaluation and use | * |  | Any feedback on the way the guideline uptake can be evaluated? And feedback on its usefulness in different settings? And uptake? |
| 1. Updating | * |  | Thoughts on how this could be done efficiently? Can implementation be included in the updating process? |
