# Supplementary material for: Strengthening prehospital clinical practice guideline implementation in South Africa: a qualitative case study
Source: BMC Health Serv Res. 2020 Apr 24;20:349. doi: 10.1186/s12913-020-05111-x (PMC7183123; doi:10.1186/s12913-020-05111-x)
Supplement: Supplementary file 2 — Additional file 2. Alternative guideline development process. [file 12913_2020_5111_MOESM2_ESM.docx]

## Additional file 2: Alternative guideline development process

| De Novo approach | AFEM Adaptation approach |
| --- | --- |
| 1. Organisation, budget, planning and training | * |
| 1. Priority setting | * |
| 1. Guideline group membership | Include advisory board (clinical and methodological) |
| 1. Establish guideline group processes | Include decision framework for using existing guidelines and recommendations. |
| 1. Identify target audience and topic selection | * |
| 1. Consumer and stakeholder involvement | * |
| 1. Conflicts of interest | * |
| 1. Question generation | Create broader questions that are transferable to key priority areas applicable and likely to be reported in guidelines |
| 1. Considering importance of outcomes and interventions, values, preferences and utilities | * |
| 1. Deciding what evidence to include and searching for evidence | Clearly defining inclusion of high-quality, up-to-date guidelines and perform comprehensive searches including guideline clearinghouses, Google and traditional databases |
| 1. Summarising evidence and considering additional information | Mapping evidence and/or guidelines by priority areas and/or questions |
| 1. Judging quality, strength or certainty of a body of evidence | Using AGREE II appraisal for guidelines and ranking included guidelines by date, relevance and overall quality |
| 1. Developing recommendations and determining their strength | Adopting, adapting or contextualising guidelines  Extract recommendations relevant to priority areas and questions  Reviewing adopted, adapted or contextualised recommendations with advisory boards |
| 1. Wording of recommendations and of considerations about implementation, feasibility and equity | Reporting original working of recommendations levels of evidence and/or strength in plain language  Considering implementation points and practice points for each recommendation that has been adopted or contextualised |
| 1. Reporting and peer review | * |
| 1. Dissemination and implementation | * |
| 1. Evaluation and use | * |
| 1. Updating | * |

*Indicates processes that are the same or implicit in both pathways.
